# Homogeneous Multifunction Devices Designing and Layered Implementing Based on Rotary Medium

**DOI:** 10.1038/s41598-018-35327-1

**Published:** 2018-11-26

**Authors:** Cheng-Fu Yang, Ming Huang, Jing-Jing Yang, Fu-Chun Mao

**Affiliations:** School of Information Science and Engineering, Wireless Innovation Lab. of Yunnan University, Kunming, 650091 P.R. China

## Abstract

Multifunctional device with homogeneous anisotropic material parameters are proposed and designed based on linear transformation optics and rotary medium. Four examples including rotating concentrator, rotating amplifying device, rotating shrinking device and rotating transparent device are reported. All of them have bi-functional effects, i.e., they possess concentrating, amplifying, shrinking and transparent effects respectively while have the fields been rotated an angle of *π*/*N* simultaneously in common, where N is the sides number of polygon. All these devices have potential applications, such as energy accumulation or controlling, military camouflage, wireless communication system and radar/antenna protection. Furthermore, alternating isotropic layered structure based on effective medium theory is utilized to remove the anisotropic property of these devices. Simulation results show that the layered structure device behaves almost as perfect as the ideal one when it has sufficient divided layers. The feasibility of designing multifunctional device by natural isotropic materials instead of metamaterials with complicated artificial composite structure would dramatically reduce the fabrication difficulty and move the device a step further towards the practical application.

## Introduction

As a mathematical approach, Transformation Optics (TO) builds a bridge of electromagnetic field distribution and material parameters distribution, and provides a powerful and convenient way for the flexible design of metamaterial devices^1–3^. The most striking device based on TO is invisible cloak^4–12^, in which arbitrary objects become invisible for electromagnetic waves. The material parameters usually possess complex properties such as inhomogeneous and anisotropic, which are difficult for the fabrication and hinder the practical application in recent time. Besides invisible cloak, concentrator^13–18^, transparent device^19–22^, amplifying device^23^, shrinking device^24–26^ and EM field rotator^27–29^ have also attracted widespread attention for their special functions and properties. For example, concentrator endows the capability to enhance the EM field distribution in the core region and has potential application in the harnessing of light in solar cells or similar devices^14,15^, while transparent device has the capability to protect an antenna or other object inside the device without affecting their performance^20,22^. Amplifying device or shrinking device can make an arbitrary shaped object virtually acts as another one with different material parameters and geometrical size, and have potential applications in military camouflage and other communication engineering fields^23,24^. EM field rotator can rotate the internal propagation direction of EM waves and can be used in the antennas and wave-guiding device that provides an approach of polarization transformation^27^. Furthermore, transformation optics has been employed to design communication devices, including beam synthesis/splitters^30,31^, antennas^32,33^ and waveguides^34^. However, it should be noted that most previous works simply investigated the single function of these devices and seldom considered the combining effectives of them.

Recently, multifunctional devices have attained remarkable attention. The first multifunctional device was proposed by Zentgraf *et al*.^35^, where an optical device acts as a lens as well as a beam-shifter simultaneously was designed and experimently verified. Later, by compositing the rotating and amplifying transformations, Zang *et al*.^36^ proposed a bi-functional device that can make an object been rotated and enlarged at the same time. However, due to the utilization of traditional radial based or folded geometrical transformations, the constitutive parameters of these bi-functional devices are inhomogeneous, anisotropic, and double negative, which hinder the practical fabrication and application. By employing the TO method and complementary medium, Mei *et al*.^37,38^ reported another novel bi-functional devices that can act as reciprocal cloak as well as transparent device, even as an illusion cloak. In another ref.^39^, Mei *et al*. developed another homogeneous illusion device that possesses transformed and shifted scattering effect simultaneously. In a more recent reference^40^, a homogeneous bi-functional device for rotator-concentrator was proposed based on multi-folded transformation optics, which has striking performance such as open-coating and remote control of EM filed. Though some of these striking devices have homogeneous parameters, the negative properties still a challenge for practical realization. The utilization of metamaterial may yield a path in manufacturing and applications, however, the narrow bandwidth and high loss of metamaterial stills a bottleneck. Thus, it is highly urgent to develop a multifunctional device with homogeneous isotropic and nonnegative material parameters.

In this letter, based on the linear transformation optics, several novel kinds of homogeneous multi-functional devices served as rotational concentrator, rotational amplifying device, rotational shrinking device and rotational transparent device are investigated. The multi-functional performances of the proposed devices are validated by the finite element method. Furthermore, layered structures based on effective medium theory^41^ are employed to remove the anisotropic properties of these devices, thus making them possess simply homogeneous, isotropic and non-negative material parameters which dramatically reduce the difficulty in practical implementation. The feasibility of designing multifunctional device by natural isotropic materials instead of metamaterial with artificial composite structures would greatly reduce the fabrication difficulties and move the devices a step further towards practical application.

## Results

### Theoretical consideration

According to transformation optics, under a space transformation from the original coordinate (*x*, *y*, *z*) to a new coordinate [*x*′(*x*, *y*, *z*), *y*′(*x*, *y*, *z*), *z*′(*x*, *y*, *z*)], the permittivity and the permeability in the transformed space are given by

1 $$\varepsilon ^{\prime} ={\rm{\Lambda }}\varepsilon {{\rm{\Lambda }}}^{T}/{\rm{\det }}\,{\rm{\Lambda }},\mu ^{\prime} ={\rm{\Lambda }}\mu {{\rm{\Lambda }}}^{T}/{\rm{\det }}\,{\rm{\Lambda }}$$

where *ε* and *μ* are the permittivity and permeability of the original space. Λ is the Jacobian transformation matrix with components ${\Lambda }_{ij}=\partial {x}_{i}^{\prime} /\partial {x}_{j},(i,j\in x,y,z)$. ${\rm{\det }}\,{\rm{\Lambda }}$ is the determinant of the matrix.

**Figure 1:**
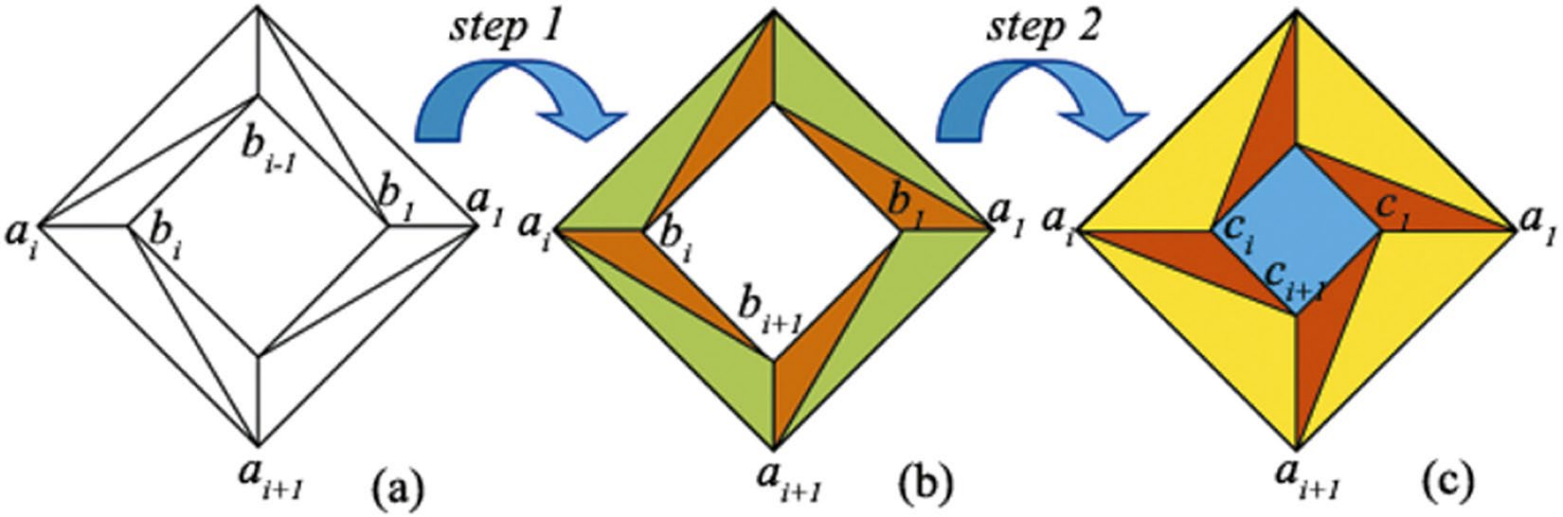
Schematic of rotating concentrator/amplifying device. (**a**) original space, (**b**) intermediate space, (**c**) physical space. The quadrilateral region *a*_*i*_*a*_*i*+1_*b*_*i*_*b*_*i*−1_ in (**a**) is firstly mapped into quadrilateral region *a*_*i*_*a*_*i*+1_*b*_*i*+1_*b*_*i*_ in (**b**), and further mapped into the quadrilateral region *a*_*i*_*a*_*i*+1_*c*_*i*+1_*c*_*i*_ in (**c**).

Firstly, we derive the material distribution of the rotational concentrator/amplifying device. Different from previous polygonal cross section metamaterial devices, we divided N regions in the original space into 2N triangles and then utilize linear transformation optics to obtain the constitutive parameters of the desired device. Figure 1 demonstrates the schematic diagram of a rotational concentrator/amplifying device.

Two steps are employed to achieve the goal. In the first step, a polygonal shell region consists of 2 N triangles in the original space are transformed into another one composes of 2N triangles in intermediate space, which forms the rotational characteristics of the device. Take the triangle region Δ*a*_*i*_*a*_*i*+1_*b*_*i*_ and region Δ*a*_*i*_*b*_*i*−1_*b*_*i*_ in the original space as example: it is transformed into triangle Δ*a*_*i*_*a*_*i*+1_*b*_*i*+1_ and Δ*a*_*i*_*b*_*i*_*b*_*i*+1_ in intermediate (rotational) space respectively.

The transformation equation of triangle Δ*a*_*i*_*a*_*i*+1_*b*_*i*_ to triangle Δ*a*_*i*_*a*_*i*+1_*b*_*i*+1_ can be expressed as:

2 $$\begin{array}{rcl}x^{\prime}  & = & {e}_{1}x+{e}_{2}y+{e}_{3},\\ y^{\prime}  & = & {f}_{1}x+{f}_{2}y+{f}_{3},\\ z^{\prime}  & = & z,\end{array}$$

where

$$[\begin{array}{cc}{e}_{1} & {f}_{1}\\ {e}_{2} & {f}_{2}\\ {e}_{3} & {f}_{3}\end{array}]={{\rm{{\rm A}}}}^{-1}[\begin{array}{cc}{x}_{{a}_{i}} & {y}_{{a}_{i}}\\ {x}_{{a}_{i+1}} & {y}_{{a}_{i+1}}\\ {x}_{{b}_{i+1}} & {y}_{{b}_{i+1}}\end{array}],\,{\rm{{\rm A}}}=[\begin{array}{ccc}{x}_{{a}_{i}} & {y}_{{a}_{i}} & 1\\ {x}_{{a}_{i+1}} & {y}_{{a}_{i+1}} & 1\\ {x}_{{b}_{i}} & {y}_{{b}_{i}} & 1\end{array}].$$

Similarly, the transformation equation of triangle Δ*a*_*i*_*b*_*i*−1_*b*_*i*_ to triangle Δ*a*_*i*_*b*_*i*_*b*_*i*+1_ can be expressed as:

3 $$\begin{array}{rcl}x^{\prime}  & = & {g}_{1}x+{g}_{2}y+{g}_{3},\\ y^{\prime}  & = & {h}_{1}x+{h}_{2}y+{h}_{3},\\ z^{\prime}  & = & z,\end{array}$$

where

$$[\begin{array}{cc}{g}_{1} & {h}_{1}\\ {g}_{2} & {h}_{2}\\ {g}_{3} & {h}_{3}\end{array}]={B}^{-1}[\begin{array}{cc}{x}_{{a}_{i}} & {y}_{{a}_{i}}\\ {x}_{{b}_{i}} & {y}_{{b}_{i}}\\ {x}_{{b}_{i+1}} & {y}_{{b}_{i+1}}\end{array}],\,B=[\begin{array}{ccc}{x}_{{a}_{i}} & {y}_{{a}_{i}} & 1\\ {x}_{{b}_{i-1}} & {y}_{{b}_{i-1}} & 1\\ {x}_{{b}_{i}} & {y}_{{b}_{i}} & 1\end{array}].$$

The Jacobian matrix of equations (2) and (3) is given by:

4 $${{\rm{\Lambda }}}_{1}=[\begin{array}{ccc}{e}_{1} & {e}_{2} & 0\\ {f}_{1} & {f}_{2} & 0\\ 0 & 0 & 1\end{array}],\,{{\rm{\Lambda }}}_{2}=[\begin{array}{ccc}{g}_{1} & {g}_{2} & 0\\ {h}_{1} & {h}_{2} & 0\\ 0 & 0 & 1\end{array}]$$

**Figure 2:**
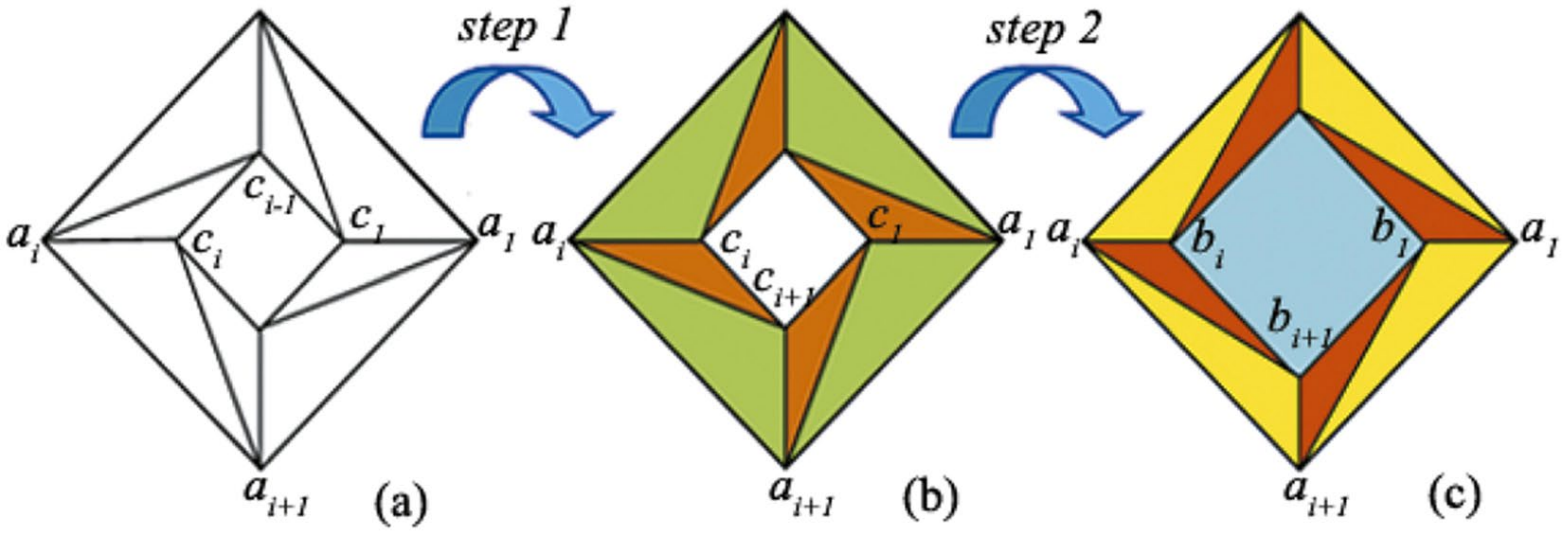
Schematic of rotational shrinking device. (**a**) The original space, (**b**) the intermediate space, (**c**) the physical space. The quadrilateral region *a*_*i*_*a*_*i*+1_*c*_*i*_*c*_*i*−1_ in (**a**) is firstly mapped into the quadrilateral region *a*_*i*_*a*_*i*+1_*c*_*i*+1_*c*_*i*_ in (**b**), and then further mapped into the quadrilateral region *a*_*i*_*a*_*i*+1_*b*_*i*+1_*b*_*i*_ in (**c**).

In the second step, the polygonal shell region in intermediate space is further expanded into a bigger one in physical space, as the triangles colored in yellow and red shown in Fig. 1(c). Thus, the core region in intermediate region is then compresses into a smaller region, as the blue colored region shown in Fig. 2(c). Take the triangle Δ*a*_*i*_*a*_*i*+1_*b*_*i*+1_ and Δ*a*_*i*_*b*_*i*_*b*_*i*+1_ in intermediate space as example, it is transformed into triangle Δ*a*_*i*_*a*_*i*+1_*c*_*i*+1_ and Δ*a*_*i*_*c*_*i*_*c*_*i*+1_ in physical space respectively.

The transformation equation of triangle Δ*a*_*i*_*a*_*i*+1_*b*_*i*+1_ to triangle Δ*a*_*i*_*a*_*i*+1_*c*_*i*+1_ can be expressed as:

5 $$\begin{array}{rcl}x^{\prime\prime}  & = & {k}_{1}x^{\prime} +{k}_{2}y^{\prime} +{k}_{3},\\ y^{\prime\prime}  & = & {l}_{1}x^{\prime} +{l}_{2}y^{\prime} +{l}_{3},\\ z^{\prime\prime}  & = & z^{\prime} ,\end{array}$$

where

$$[\begin{array}{cc}{k}_{1} & {l}_{1}\\ {k}_{2} & {l}_{2}\\ {k}_{3} & {l}_{3}\end{array}]={C}^{-1}[\begin{array}{cc}{x}_{{a}_{i}} & {y}_{{a}_{i}}\\ {x}_{{a}_{i+1}} & {y}_{{a}_{i+1}}\\ {x}_{{c}_{i+1}} & {y}_{{c}_{i+1}}\end{array}],C=[\begin{array}{ccc}{x}_{{a}_{i}} & {y}_{{a}_{i}} & 1\\ {x}_{{a}_{i+1}} & {y}_{{a}_{i+1}} & 1\\ {x}_{{b}_{i}} & {y}_{{b}_{i}} & 1\end{array}].$$

And the triangle Δ*a*_*i*_*b*_*i*_*b*_*i*+1_ to triangle Δ*a*_*i*_*c*_*i*_*c*_*i*+1_ can be expressed as:

6 $$\begin{array}{rcl}x^{\prime\prime}  & = & {m}_{1}x^{\prime} +{m}_{2}y^{\prime} +{m}_{3},\\ y^{\prime\prime}  & = & {n}_{1}x^{\prime} +{n}_{2}y^{\prime} +{n}_{3},\\ z^{\prime\prime}  & = & z^{\prime} ,\end{array}$$

where

$$[\begin{array}{cc}{m}_{1} & {n}_{1}\\ {m}_{2} & {n}_{2}\\ {m}_{3} & {n}_{3}\end{array}]={D}^{-1}[\begin{array}{cc}{x}_{{a}_{i}} & {y}_{{a}_{i}}\\ {x}_{{c}_{i}} & {y}_{{c}_{i}}\\ {x}_{{c}_{i+1}} & {y}_{{c}_{i+1}}\end{array}],\,D=[\begin{array}{ccc}{x}_{{a}_{i}} & {y}_{{a}_{i}} & 1\\ {x}_{{b}_{i}} & {y}_{{b}_{i}} & 1\\ {x}_{{b}_{i+1}} & {y}_{{b}_{i+1}} & 1\end{array}].$$

The Jacobian matrix of equations (5) and (6) is given by:

7 $${{\rm{\Lambda }}}_{3}=[\begin{array}{ccc}{k}_{1} & {k}_{2} & 0\\ {l}_{1} & {l}_{2} & 0\\ 0 & 0 & 1\end{array}],\,{{\rm{\Lambda }}}_{4}=[\begin{array}{ccc}{m}_{1} & {m}_{2} & 0\\ {n}_{1} & {n}_{2} & 0\\ 0 & 0 & 1\end{array}]$$

Then the constitutive permittivity and permeability of the triangle Δ*a*_*i*_*a*_*i*+1_*c*_*i*+1_ can be obtained by:

8 $$\begin{array}{rcl}\mu {^{\prime} }_{outer} & = & \mu [\begin{array}{cc}({M}_{1}^{2}+{M}_{2}^{2})/({M}_{1}{N}_{2}-{M}_{2}{N}_{1}) & ({M}_{1}{N}_{1}+{M}_{2}{N}_{2})/({M}_{1}{N}_{2}-{M}_{2}{N}_{1})\\ ({M}_{1}{N}_{1}+{M}_{2}{N}_{2})/({M}_{1}{N}_{2}-{M}_{2}{N}_{1}) & ({N}_{1}^{2}+{N}_{2}^{2})/({M}_{1}{N}_{2}-{M}_{2}{N}_{1})\end{array}],\\ \varepsilon {^{\prime} }_{outer} & = & \varepsilon /({M}_{1}{N}_{2}-{M}_{2}{N}_{1}).\end{array}$$

where *M*_1_ = *e*_1_*k*_1_ + *e*_2_*l*_1_, *M*_2_ = *e*_1_*k*_2_ + *e*_2_*l*_2_, *N*_1_ = *f*_1_*k*_1_ + *f*_2_*l*_1_, *N*_2_ = *f*_1_*k*_2_ + *f*_2_*l*_2_.

And the constitutive permittivity and permeability of the triangle Δ*a*_*i*_*c*_*i*_*c*_*i*+1_ can be obtained by:

9 $$\begin{array}{rcl}\mu {^{\prime} }_{inner} & = & \mu [\begin{array}{cc}({M}_{3}^{2}+{M}_{4}^{2})/({M}_{3}{N}_{4}-{M}_{4}{N}_{3}) & ({M}_{3}{N}_{3}+{M}_{4}{N}_{4})/({M}_{3}{N}_{4}-{M}_{4}{N}_{3})\\ ({M}_{3}{N}_{3}+{M}_{4}{N}_{4})/({M}_{3}{N}_{4}-{M}_{4}{N}_{3}) & ({N}_{3}^{2}+{N}_{4}^{2})/({M}_{3}{N}_{4}-{M}_{4}{N}_{3})\end{array}],\\ \varepsilon {^{\prime} }_{inner} & = & \varepsilon /({M}_{3}{N}_{4}-{M}_{4}{N}_{3}).\end{array}$$

where *M*_3_ = *g*_1_*m*_1_ + *g*_2_*n*_1_, *M*_4_ = *g*_1_*m*_2_ + *g*_2_*n*_2_, *N*_3_ = *h*_1_*m*_1_ + *h*_2_*n*_1_, *N*_4_ = *h*_1_*m*_2_ + *h*_2_*n*_2_.

For the core region of the rotational device, a big square with circum-radius b is compressed into a small square with circum-radius c, and the transformation equations can be expressed as:

10 $$\begin{array}{c}x^{\prime} =\frac{b}{c}x,\\ y^{\prime} =\frac{b}{c}y,\\ z^{\prime\prime} =z^{\prime} .\end{array}$$

Thus, the constitutive parameters of this core region will become:

11 $$\begin{array}{c}\mu {^{\prime} }_{core}=\mu ,\\ \varepsilon {^{\prime} }_{core}={(b/c)}^{2}\varepsilon ,\end{array}$$

where *μ* and *ε* are the relative permeability and permittivity of the air, and are set to as *μ* = *ε* = 1 during the simulation. Since the core region of the device in physical space is transformed from a larger one in virtual space, an object in this region will looks like another bigger one visually.

Secondly, we present the material distribution of rotational shrinking device. Similar as the rotational amplifying device, two steps are needed to obtain the rotational shrinking device. Figure 2 displays the schematic diagram of the rotational shrinking device. The goal of the first step is to obtain the rotational performance of the device, which can be easily obtained by transform triangle Δ*a*_*i*_*a*_*i*+1_*c*_*i*_ and Δ*a*_*i*_*c*_*i*−1_*c*_*i*_ in original space into triangle Δ*a*_*i*_*a*_*i*+1_*c*_*i*+1_ and Δ*a*_*i*_*c*_*i*_*c*_*i*+1_ in intermediate (rotational) space, as shown in Fig. 2(a) and (b). From observing Fig. 1 and 2, it can be found that step one is almost identical. Thus, by simply replacing the vertexes’ coordinates of polygons, one can easily obtain the transformation equations and corresponding Jacobean matrixes as given in Eqs (2)–(4), where

$$[\begin{array}{cc}{e}_{1} & {f}_{1}\\ {e}_{2} & {f}_{2}\\ {e}_{3} & {f}_{3}\end{array}]={{\rm{{\rm A}}}}^{-1}[\begin{array}{cc}{x}_{{a}_{i}} & {y}_{{a}_{i}}\\ {x}_{{a}_{i+1}} & {y}_{{a}_{i+1}}\\ {x}_{{c}_{i+1}} & {y}_{{c}_{i+1}}\end{array}],\,{\rm{{\rm A}}}=[\begin{array}{ccc}{x}_{{a}_{i}} & {y}_{{a}_{i}} & 1\\ {x}_{{a}_{i+1}} & {y}_{{a}_{i+1}} & 1\\ {x}_{{c}_{i}} & {y}_{{c}_{i}} & 1\end{array}],[\begin{array}{cc}{g}_{1} & {h}_{1}\\ {g}_{2} & {h}_{2}\\ {g}_{3} & {h}_{3}\end{array}]={B}^{-1}[\begin{array}{cc}{x}_{{a}_{i}} & {y}_{{a}_{i}}\\ {x}_{{c}_{i}} & {y}_{{c}_{i}}\\ {x}_{{c}_{i+1}} & {y}_{{c}_{i+1}}\end{array}],\,B=[\begin{array}{ccc}{x}_{{a}_{i}} & {y}_{{a}_{i}} & 1\\ {x}_{{c}_{i-1}} & {y}_{{c}_{i-1}} & 1\\ {x}_{{c}_{i}} & {y}_{{c}_{i}} & 1\end{array}].$$

Similarly, the transformation equations and corresponding Jacobean matrix can be given as Eqs (5)–(7) for step 2, by just replacing *b*_*i*_, *b*_*i*+1_ with *c*_*i*_, *c*_*i*+1_, i.e.

$$[\begin{array}{cc}{k}_{1} & {l}_{1}\\ {k}_{2} & {l}_{2}\\ {k}_{3} & {l}_{3}\end{array}]={C}^{-1}[\begin{array}{cc}{x}_{{a}_{i}} & {y}_{{a}_{i}}\\ {x}_{{a}_{i+1}} & {y}_{{a}_{i+1}}\\ {x}_{{b}_{i+1}} & {y}_{{b}_{i+1}}\end{array}],\,C=[\begin{array}{ccc}{x}_{{a}_{i}} & {y}_{{a}_{i}} & 1\\ {x}_{{a}_{i+1}} & {y}_{{a}_{i+1}} & 1\\ {x}_{{c}_{i}} & {y}_{{c}_{i}} & 1\end{array}],[\begin{array}{cc}{m}_{1} & {n}_{1}\\ {m}_{2} & {n}_{2}\\ {m}_{3} & {n}_{3}\end{array}]={D}^{-1}[\begin{array}{cc}{x}_{{a}_{i}} & {y}_{{a}_{i}}\\ {x}_{{b}_{i}} & {y}_{{b}_{i}}\\ {x}_{{b}_{i+1}} & {y}_{{b}_{i+1}}\end{array}],\,D=[\begin{array}{ccc}{x}_{{a}_{i}} & {y}_{{a}_{i}} & 1\\ {x}_{{c}_{i}} & {y}_{{c}_{i}} & 1\\ {x}_{{c}_{i+1}} & {y}_{{c}_{i+1}} & 1\end{array}].$$

Thus, the constitutive parameters of the outer and inner triangles can be calculated by Eqs (8) and (9).

However, for the core region of step 2, the small square region with circum-radius of c is expanded into a bigger one with circum-radius of b, and the transformation equations can be expressed as:

12 $$\begin{array}{c}x^{\prime} =\frac{c}{b}x,\\ y^{\prime} =\frac{c}{b}y,\\ z^{\prime\prime} =z^{\prime} .\end{array}$$

Thus, the constitutive parameters of the core region will become as:

13 $$\begin{array}{c}\mu {^{\prime} }_{core}=1,\\ \varepsilon {^{\prime} }_{core}={(c/b)}^{2}\varepsilon .\end{array}$$

**Figure 3:**
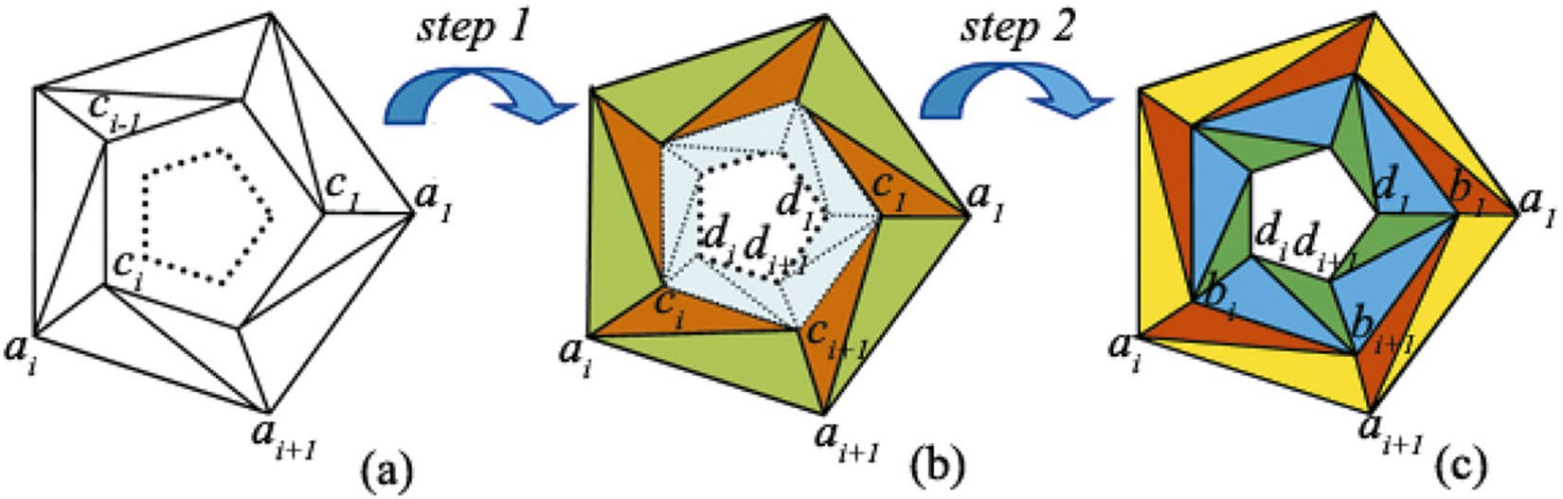
Schematic diagram of rotational transparent device. (**a**) The original space, (**b**) the intermediate space, (**c**) the physical space. The quadrilateral region *a*_*i*_*a*_*i*+1_*c*_*i*_*c*_*i*−1_ in (**a**) is firstly mapped into the quadrilateral region *a*_*i*_*a*_*i*+1_*c*_*i*+1_*c*_*i*_ in (**b**), and then further mapped into the quadrilateral region *a*_*i*_*a*_*i*+1_*b*_*i*+1_*b*_*i*_ in (**c**). Simultaneously, the quadrilateral region *d*_*i*_*d*_*i*+1_*c*_*i*+1_*c*_*i*_ in (**b**) is mapped into the quadrilateral region *d*_*i*_*d*_*i*+1_*b*_*i*+1_*b*_*i*_ in (**c**).

Next, we further derive the material distribution for rotational transparent device. To obtain a rotational transparent device, two steps are also needed, as shown in Fig. 3. The first step is to obtain a rotational medium that is exactly the same as the procedure of rotational shrinking device. Thus, we omit the description of this transformation.

However, in step 2, two sub stages are employed. In sub stage 1, a bigger polygonal shell region (as the green and orange colored regions shown in Fig. 3(b)) is compresses into a smaller one (as the yellow and red colored regions shown in Fig. 3(c)). This procedure is also identical to the rotational shrinking device, and the constitutive parameters can be easily obtained by substituting the vertexes values of the polygons into Eqs (8) and (9).

In sub stage 2, a smaller polygonal shell which is bordered between d < r < c (as the gray colored region shown in Fig. 3(b)) is expanded to a big one that is bordered between d < r < b (as the dark blue and dark green colored regions shown in Fig. 3(c)). Take *d*_*i*_*c*_*i*_*c*_*i*+1_*d*_*i*+1_ as example, the outer triangle Δ*d*_*i*_*c*_*i*_*c*_*i*+1_ is transformed into triangle Δ*d*_*i*_*b*_*i*_*b*_*i*+1_, while the inner triangle Δ*d*_*i*_*d*_*i*+1_*c*_*i*+1_ is transformed into triangle Δ*d*_*i*_*d*_*i*+1_*b*_*i*+1_.

The transformation equation of triangle Δ*d*_*i*_*c*_*i*_*c*_*i*+1_ to triangle Δ*d*_*i*_*b*_*i*_*b*_*i*+1_ can be expressed as:

14 $$\begin{array}{c}x^{\prime\prime} ={o}_{1}x^{\prime} +{o}_{2}y^{\prime} +{o}_{3},\\ y^{\prime\prime} ={p}_{1}x^{\prime} +{p}_{2}y^{\prime} +{p}_{3},\\ z^{\prime\prime} =z^{\prime} ,\end{array}$$

where

$$[\begin{array}{cc}{o}_{1} & {p}_{1}\\ {o}_{2} & {p}_{2}\\ {o}_{3} & {p}_{3}\end{array}]={E}^{-1}[\begin{array}{cc}{x}_{{d}_{i}} & {y}_{{d}_{i}}\\ {x}_{{b}_{i}} & {y}_{{b}_{i}}\\ {x}_{{b}_{i+1}} & {y}_{{b}_{i+1}}\end{array}],E=[\begin{array}{ccc}{x}_{{d}_{i}} & {y}_{{d}_{i}} & 1\\ {x}_{{c}_{i}} & {y}_{{c}_{i}} & 1\\ {x}_{{c}_{i+1}} & {y}_{{c}_{i+1}} & 1\end{array}].$$

Then the constitutive parameters of triangle Δ*d*_*i*_*b*_*i*_*b*_*i*+1_ can be obtained as

15 $$\begin{array}{rcl}\mu {^{\prime} }_{in\_outer} & = & \mu [\begin{array}{cc}({o}_{1}^{2}+{o}_{2}^{2})/({o}_{1}{p}_{2}-{o}_{2}{p}_{1}) & ({o}_{1}{p}_{1}+{o}_{2}{p}_{2})/({o}_{1}{p}_{2}-{o}_{2}{p}_{1})\\ ({o}_{1}{p}_{1}+{o}_{2}{p}_{2})/({o}_{1}{p}_{2}-{o}_{2}{p}_{1}) & ({p}_{1}^{2}+{p}_{2}^{2})/({o}_{1}{p}_{2}-{o}_{2}{p}_{1})\end{array}]\\ \varepsilon {^{\prime} }_{in\_outer} & = & \varepsilon /({o}_{1}{p}_{2}-{o}_{2}{p}_{1})\end{array}$$

The transformation equation of triangle Δ*d*_*i*_*d*_*i*+1_*c*_*i*+1_ to triangle Δ*d*_*i*_*d*_*i*+1_*b*_*i*+1_ can be expressed as:

16 $$\begin{array}{c}x^{\prime\prime} ={q}_{1}x^{\prime} +{q}_{2}y^{\prime} +{q}_{3},\\ y^{\prime\prime} ={r}_{1}x^{\prime} +{r}_{2}y^{\prime} +{r}_{3},\\ z^{\prime\prime} =z^{\prime} ,\end{array}$$

where

$$[\begin{array}{cc}{q}_{1} & {r}_{1}\\ {q}_{2} & {r}_{2}\\ {q}_{3} & {r}_{3}\end{array}]={F}^{-1}[\begin{array}{cc}{x}_{{d}_{i}} & {y}_{{d}_{i}}\\ {x}_{{d}_{i+1}} & {y}_{{d}_{i+1}}\\ {x}_{{b}_{i+1}} & {y}_{{b}_{i+1}}\end{array}],F=[\begin{array}{ccc}{x}_{{d}_{i}} & {y}_{{d}_{i}} & 1\\ {x}_{{d}_{i+1}} & {y}_{{d}_{i+1}} & 1\\ {x}_{{c}_{i+1}} & {y}_{{c}_{i+1}} & 1\end{array}].$$

Then, the constitutive permittivity and permeability of the triangle Δ*d*_*i*_*d*_*i*+1_*b*_*i*+1_ can be obtained by:

17 $$\begin{array}{c}\mu {^{\prime} }_{in\_outer}=\mu [\begin{array}{cc}({q}_{1}^{2}+{q}_{2}^{2})/({q}_{1}{r}_{2}-{q}_{2}{r}_{1}) & ({q}_{1}{r}_{1}+{q}_{2}{r}_{2})/({q}_{1}{r}_{2}-{q}_{2}{r}_{1})\\ ({q}_{1}{r}_{1}+{q}_{2}{r}_{2})/({q}_{1}{r}_{2}-{q}_{2}{r}_{1}) & ({r}_{1}^{2}+{r}_{2}^{2})/({q}_{1}{r}_{2}-{q}_{2}{r}_{1})\end{array}],\\ \varepsilon {^{\prime} }_{in\_outer}=\varepsilon /({q}_{1}{r}_{2}-{q}_{2}{r}_{1}).\end{array}$$

For the regular n-sided polygonal multi-functional devices, polygon A, B, C and D share the same center at origin (0, 0). Thus, the general expression of the *ith* vertex of polygon A, B, C and D can be defined as

18 $$\begin{array}{rcl}{x}_{ai} & = & a\,\cos \,[(i-1)2\pi /N],{y}_{ai}=a\,\sin \,[(i-1)2\pi /N],\\ {x}_{bi} & = & b\,\cos \,[(i-1)2\pi /N],{y}_{bi}=b\,\sin \,[(i-1)2\pi /N],\\ {x}_{ci} & = & c\,\cos \,[(i-1)2\pi /N],{y}_{ci}=c\,\sin \,[(i-1)2\pi /N],\\ {x}_{di} & = & d\,\cos \,[(i-1)2\pi /N],{y}_{di}=d\,\sin \,[(i-1)2\pi /N].\end{array}$$

where $1\le i\le N$, and *a*, *b*, *c*, *d* are the circum-radius of the *ith* vertex of polygon A, B, C and D respectively.

In order to confirm the effectiveness of the above mentioned material distributions of rotary mediums, numerical simulations are carried by finite element solve COMSOL Multiphysics under transverse electric (TE) wave irradiation with a frequency of 10 GHz.

### Rotating concentrator

**Figure 4:**
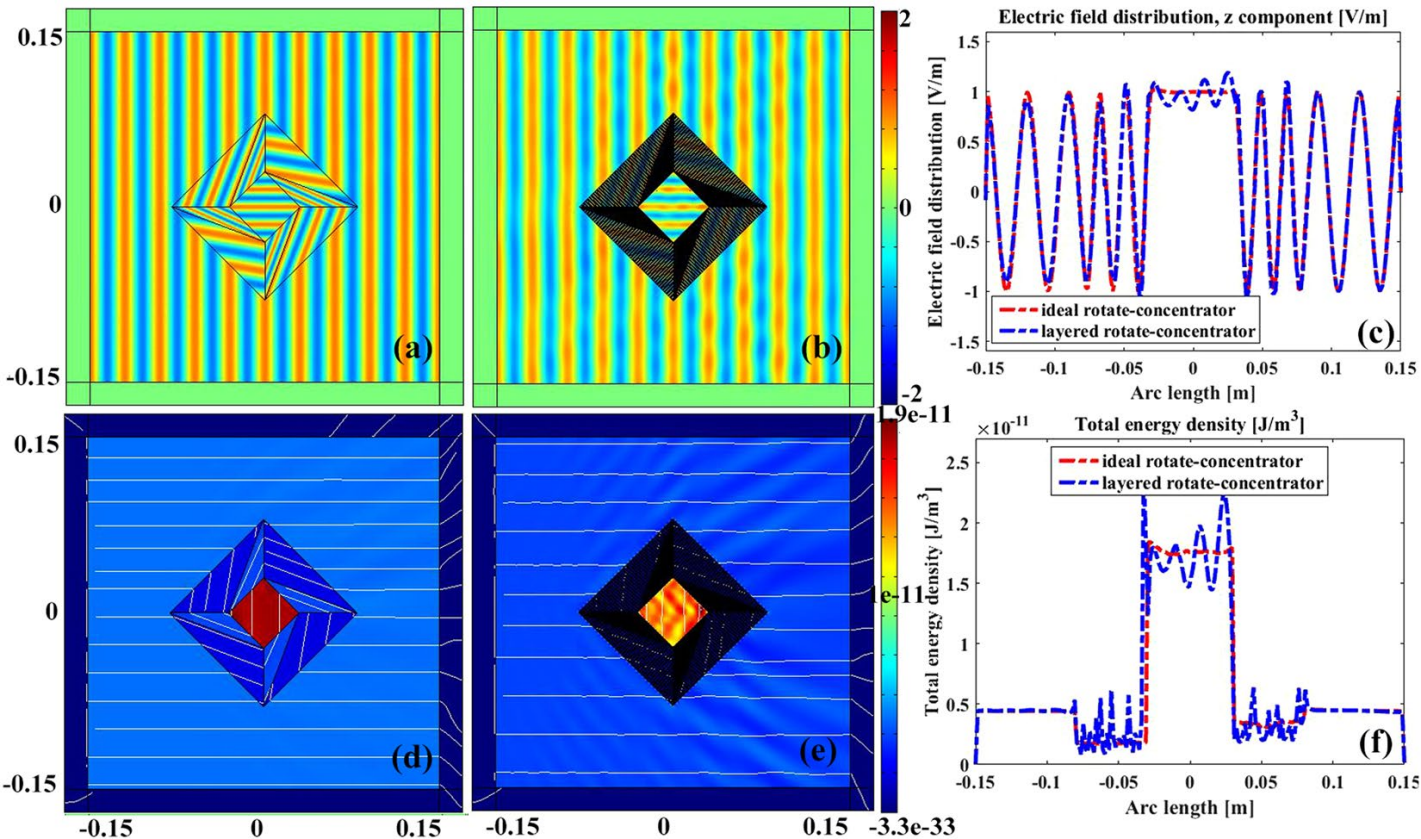
The electric field distribution in the vicinity of an ideal rotating concentrator (**a**), and layered rotating concentrator (**b**), respectively. The normalized far field distribution of an ideal rotating concentrator (**d**), layered rotating concentrator (**e**), respectively. The near field distribution (**c**) and normalized far field distribution (**f**) at the observing line y = 0 for the both ideal and the layered rotating concentrator.

Firstly, the working functionality of rotating concentrator device is demonstrated in Fig. 4. The geometric parameters of the device are set as *a* = 8 *cm*, *b* = 6 *cm*, and *c* = 3 *cm*, where *a*, *b*, *c* are the circum-radius of polygon A, B and C respectively. From Fig. 4(a), it can be clearly seen that the EM field in the core region is rotated by an angle of *π*/4, and is perpendicular to the incident wave direction. It is also observed that the rotational concentrator device itself is invisible since there is no scattering field outside the device. The EM field amplitude of the proposed device is equivalent to that in free space near the outer boundary of concentrator, which can be clearly seen from Fig. 4(c) where the electric field distribution is calculated at the observing line of y = 0. Figure 4(d) demonstrates the total energy density distribution of the proposed rotational concentrator, where the white colored lines indicate the power flow direction. Obviously, the energy is mainly concentrated in the core region of the device, which implies the potential application of the proposed device in energy accumulation or store. Similar conclusion can be drawn from Fig. 4(f) where the total energy distribution is calculated from the observing line of y = 0.

Since the constitutive parameters of the device are wholly depended on the vertexes of the polygons that are constants, they are homogeneous and anisotropic. Although homogeneous and anisotropic material makes the proposed device closer to practice, it is still difficult to fabricate. Fortunately, the anisotropic properties can be removed by utilizing alternating layered isotropic dielectrics based on effective medium theory. The corresponding rotation angle between the layer and horizontal direction can be uniquely determined by

19 $${\theta }_{i}={\tan }^{-1}[2{\mu }_{xy}^{i}/({\mu }_{xx}^{i}-{\mu }_{yy}^{i})]/2$$

20 $${\mu }_{i}^{A,B}={\mu }_{i}^{u}\pm \sqrt{{\mu }_{i}^{u}({\mu }_{i}^{u}-{\mu }_{i}^{v})},\,{\varepsilon }_{i}^{A,B}={\varepsilon }_{zz},$$

where $${\mu }_{i}^{u,v}=[{\mu }_{xx}^{i}+{\mu }_{yy}^{i}\pm \sqrt{{({\mu }_{xx}^{i}-{\mu }_{yy}^{i})}^{2}+4{({\mu }_{xy}^{i})}^{2}}]/2$$.

After detailed calculation, the layered material parameters of the rotational device are as follows:

For the outer triangles: ${{\varepsilon }_{i}}^{A}=21.7707$, ${{\varepsilon }_{i}}^{B}=0.0459$, *ε*_*zz*_ = 0.4. For the inner triangles: ${{\varepsilon }_{i}}^{A}=67.9264,\,{{\varepsilon }_{i}}^{B}=$ $0.0147$, *ε*_*zz*_ = 0.8. The rotated angle between the layer and horizontal direction can be uniquely determined by Equation (19) for each triangle.

The distributions of electric field and total energy density of the layered rotating concentrator device are shown in Fig. 4(b) and (e), where the number of layers is chosen as N = 30, i.e., 30 layers for both medium A and medium B. It can be observed that disturbances occurr in both near electric field and total energy density of the proposed layered device. However, it is small and negligible, as shown in Fig. 4(c) and (f). The main reasons for the disturbance may be come from the meshing sizes of computation and/or the divided layers, and may be reduced by increasing the meshing grids or layered numbers, but it will take more computation time to converge to an optimal solution.

### Rotating amplifying device

**Figure 5:**
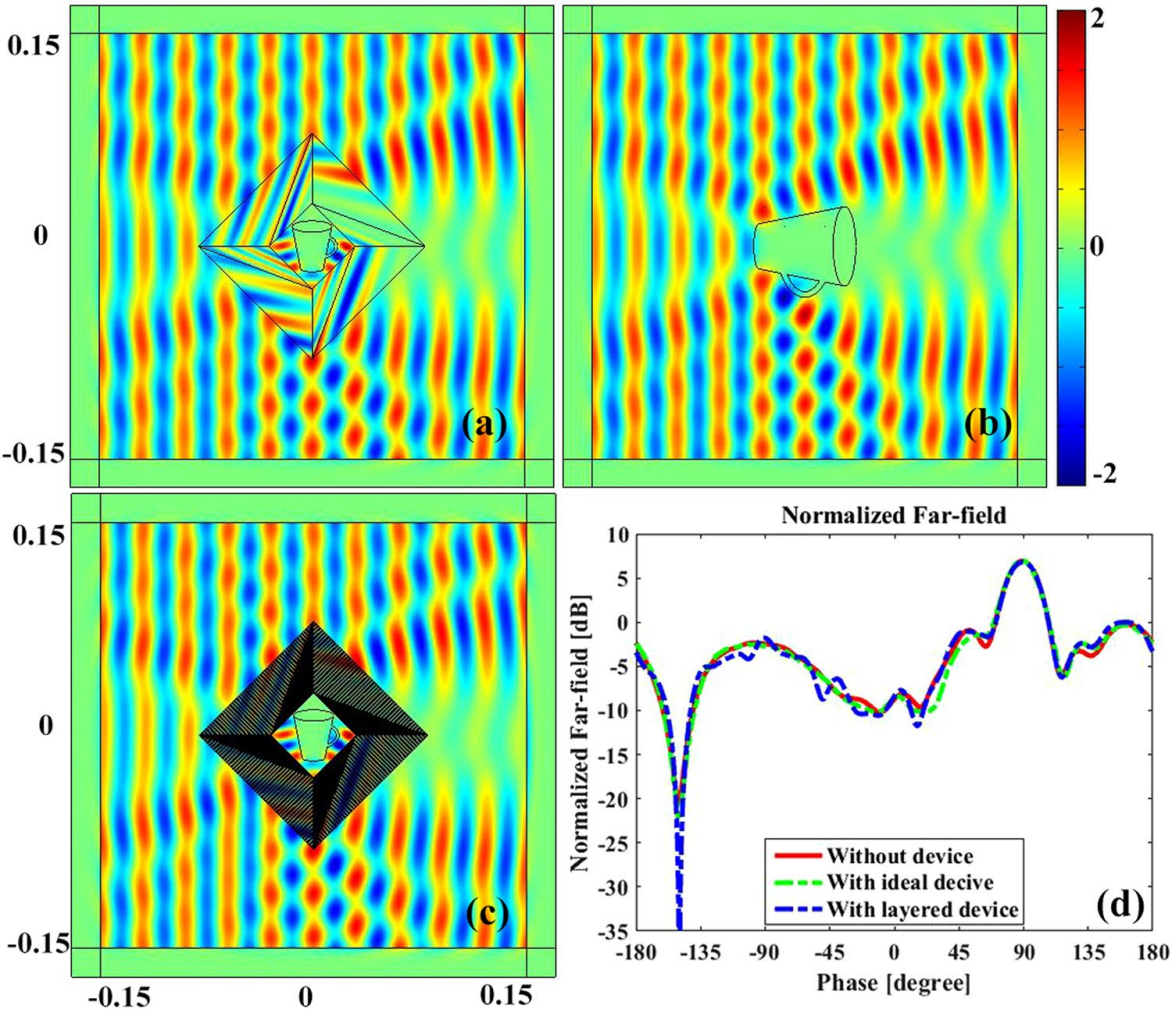
The electric field distribution of the proposed rotary amplifying device. (**a**) The near field distribution of a small cup with material parameter of *ε*_*r*_ = −4, *μ*_*r*_ = 1 coated by the ideal rotating medium. (**b**) The near field distribution of a bare cup with material parameter of *ε*_*r*_ = −1, *μ*_*r*_ = 1 in free space with a rotating angle of *π*/4. (**c**) The field distribution of a small cup with material parameter of *ε*_*r*_ = −4, *μ*_*r*_ = 1 surrounded by the layered rotary amplifying device. (**d**) the normalized far field distribution of (**a**), (**b**) and (**c**).

Secondly, we investigate the amplifying effect of the rotating concentrator. The core region of a concentrator is compressed from a bigger one, making it acts as an amplifying device as well as a concentrator or invisible cloak. Figure 5 demonstrates the functionality of the proposed rotating amplifying device. As an example, a small cup with the material parameter of *ε*_*r*_ = −4, *μ*_*r*_ = 1 is put inside the core region of the proposed device, as shown in Fig. 5(a), which also displays the electric field distribution in the vicinity of the device. For compotation, a bigger cup with material parameter of *ε*_*r*_ = −1, *μ*_*r*_ = 1 is lied in free space and rotated by an angle of *π*/4 to that of the proposed device. The electric field in the vicinity of the cup is illustrated in Fig. 5(b). It is observed that the scattering fields of Fig. 5(a) and (b) are almost identical, which confirms the amplifying capability as well as rotating effect of the device. Furthermore, it also demonstrates the performance to make an object looks like another one with both different size and material parameter. It is believed that such characteristic may have potential applications in military camouflage or other communication engineering. For example, amplifying a missile or fighter equipment to deter enemy, or miniaturizing antennas while achieve high gains.

Layered structure based on effective medium theory is used to remove the anisotropic property of the proposed rotating amplifying device, where the layered material parameters are identical to that of rotating concentrator. Figure 5(c) demonstrates the electric filed distribution in the vicinity of the layered device, where the number of divided layers is chosen as N = 30, i.e. 30 layers for both medium A and medium B. For the outer triangles, parameters of the isotropic materials are ${\varepsilon }_{i}^{A}=21.7707$, ${\varepsilon }_{i}^{B}=0.0459$, *ε*_*zz*_ = 0.4, for the inner triangles: ${\varepsilon }_{i}^{A}=67.9264$, ${\varepsilon }_{i}^{B}=0.0147$, *ε*_*zz*_ = 0.8. Making a comparison from Fig. 5(a)–(c), it can be clearly found that the scattering patterns of them are almost identical, which confirm the effectiveness of the layered structure device as well as an ideal one that successfully rotate and amplify target object coated by the device. To make a quantitatively comparison of them, the normalized far field is calculated, as shown in Fig. 5(d), where the red colored line, green colored line and blue colored line indicate the field of Fig. 5(b),(a) and (c) respectively. It is observed that they are almost overlapped together, which further confirms the effectiveness of the proposed rotating amplifying device.

### Rotating shrinking device

**Figure 6:**
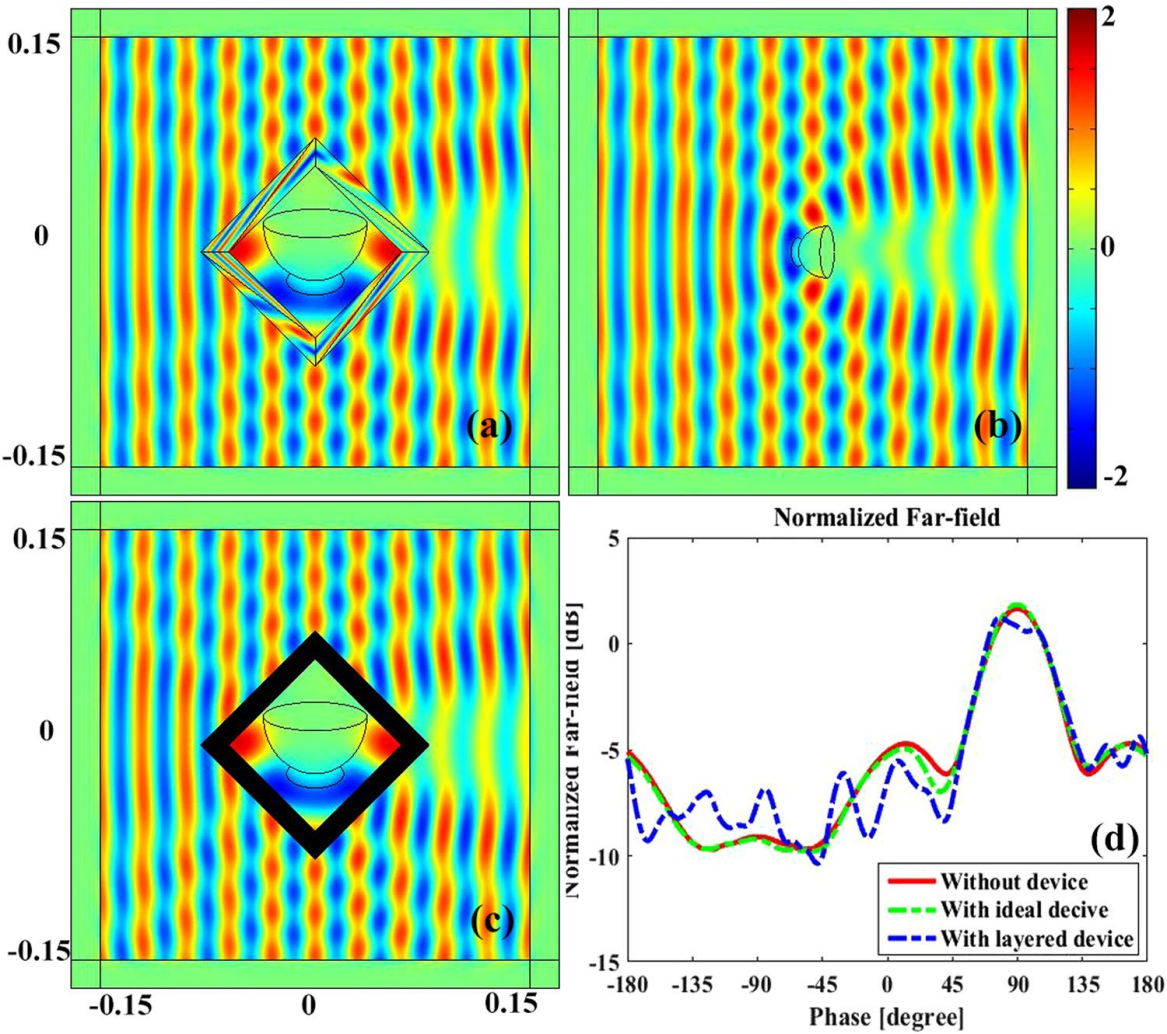
The electric field distribution of the proposed rotary shrinking device. (**a**) The near field distribution of a bowl with material parameter of *ε*_*r*_ = −0.25, *μ*_*r*_ = 1 coated by the ideal rotating medium. (**b**) The near field distribution of a bare dielectric bowl with material parameter of *ε*_*r*_ = −1, *μ*_*r*_ = 1 in free space with a rotating angle of *π*/4. (**c**) The field distribution of a bowl with material parameter of *ε*_*r*_ = −0.25, *μ*_*r*_ = 1 surrounded by the layered rotary amplifying device. (**d**) The normalized far field distribution of (**a**), (**b**) and (**c**).

In the following, we demonstrate another rotating medium that makes a bigger object looks like another smaller one, and rotates an angle simultaneously. The geometric parameters of the device are set as *a* = 8 *cm*, *b* = 6 *cm*, and *c* = 3 *cm*, where *a*, *b*, *c* are the circum-radius of polygon A, B and C respectively. Figure 6(a) shows the electric field distribution in the vicinity of the ideal rotating shrinking device where a big bowl with material parameter of *ε*_*r*_ = −0.25, *μ*_*r*_ = 1 is enclosed by the rotating medium. For comparison, a small bowl with material parameter of *ε*_*r*_ = −1, *μ*_*r*_ = 1 is put in free space and rotated with an angle of *π*/4, as shown in Fig. 6(b). Obviously, the scattering fields of Fig. 6(a) and (b) are almost identical, which confirm the effectiveness of proposed rotating medium to shrink target object and rotate an angle visually at the same time.

Next, alternating layered structure is adopted to remove the anisotropic property. The alternating isotropic material medium of the outer triangles are obtained as ${\varepsilon }_{i}^{A}=21.7707$, ${\varepsilon }_{i}^{B}=0.0459$, *ε*_*zz*_ = 1.25, while the inner triangles are ${\varepsilon }_{i}^{A}=67.9264$, ${\varepsilon }_{i}^{{\rm{B}}}=0.0147$, *ε*_*zz*_ = 1.25. Similarly, the rotational angle between the alternative layer and horizontal direction can be easily obtained from Equation (19).

It is clear that all isotropic mediums utilized are nonnegative, which allows the device to be fabricated by natural materials. 30 layers for both isotropic medium A and B are utilized in the simulation. Figure 6(c) demonstrates the electric field distribution in the vicinity of the alternative layered structure device. From Fig. 6(a–c), it is found that the electric field distributions around the devices are nearly identical, which verify the effectiveness of the devices to shrink target object and to rotate an angle simultaneously. Moreover, normalized far fields of the ideal device, bare rotating bowls and alternative layered device are carried out to make a quantitative comparison, as the green colored dashed line, red colored line and blue colored dashed line shown in Fig. 6(d), respectively. They agree well with each other which confirm the correctness of the designing and the effectiveness of the proposed device. The small field disturbance of the layered structure device comes from the mesh size and divided layer which can be greatly reduced by using finer mesh and more stratification, but will take more computation time to obtain an optimal solution. Such rotating shrinking device may have potential applications in military camouflage or communication engineering, such as reduce the RCS of Radar or other target object.

### Rotating transparent device

**Figure 7:**
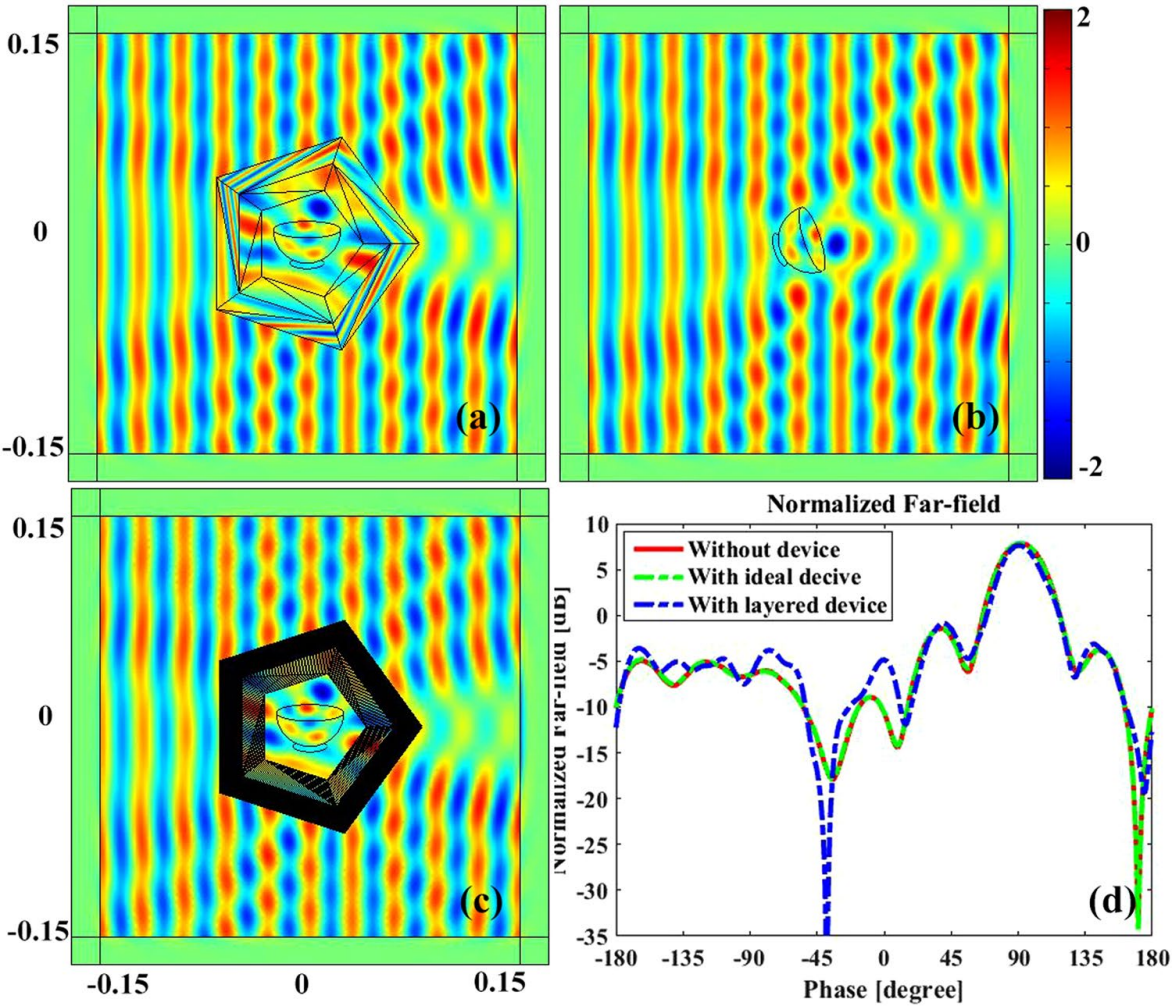
The electric field distribution of the proposed rotary transparent device. (**a**) The near field distribution of a bowl coated by the ideal rotating medium. (**b**) The near field distribution of a bare dielectric bowl. (**c**) The field distribution of the layered rotary transparent device. (**d**) The normalized far field distribution of (**a**), (**b**) and (**c**). The material parameter of the dielectric bowl is *ε*_*r*_ = 2.65 + 0.01j, *μ*_*r*_ = 1.

Finally, we utilize rotating medium to design a transparent device which possesses rotation and transparency properties simultaneously. Regularly 5-sided polygonal cross section with circum-radius of *a* = 8 *cm*, *b* = 6 *cm*, *c* = 5 *cm*, and *d* = 4 *cm* are employed to design the device. Figure 7(a) demonstrates the electric field distribution in the vicinity of an ideal rotating transparent device, where a bowl with material parameter of *ε*_*r*_ = 2.65 + 0.01j, *μ*_*r*_ = 1 is coated by the rotating medium. In Fig. 7(b), a bowl with identical size and material parameter is set in the free space but rotated with an angle of *π*/5. By comparing the electric field distributions of Fig. 7(a) and (b), it is found that they are identical, which confirms the transparent and rotary performance of the proposed device.

Similarly, we exploit alternative isotropic material layers to remove the anisotropic property of the proposed ideal rotating transparent device. After detailed calculation, the material parameters of the isotropic medium are obtained as follows:

For the outer triangles of the outer shell: ${\varepsilon }_{i}^{A}=25.4275$, ${\varepsilon }_{i}^{{\rm{B}}}=0.0393$, *ε*_*zz*_ = 1.5.

For the inner triangles of the outer shell: ${\varepsilon }_{i}^{A}=50.0310$, ${\varepsilon }_{i}^{{\rm{B}}}=0.02$, *ε*_*zz*_ = 1.25.

For the outer triangles of the inner shell: ${{\varepsilon }_{i}}^{A}=3.4583$,${{\varepsilon }_{i}}^{{\rm{B}}}=0.2892$,*ε*_*zz*_ = 0.4167.

For the inner triangles of the inner shell: ${\varepsilon }_{i}^{A}=4.4467$, ${\varepsilon }_{i}^{{\rm{B}}}=0.2249$, *ε*_*zz*_ = 0.5.

The rotational angles also can be easily calculated from Equation (19). The number of divided layer is chosen as N = 30 for all alternative isotropic medium. Figure 7(c) demonstrates the near electric field distribution around the alternative layered structure device, which is also almost identical to that of the ideal one and confirms the correctness and effectiveness of the layered approach. We further calculate the normalized far field to make a quantitative comparison of them, as shown in Fig. 7(d), where green colored dashed line, red colored line and blue colored dashed line indicate the field distribution of the ideal rotary transparent device, the bare dielectric object and the layered structure device, respectively. Apparently, they agree well with each other. It is also found that the normalized far field of the layered structure device appeals subtle disturbance. However, it is very small and negligible. The almost perfect performance of the rotating transparent device has potential application, such as antenna protection and polarization transformation.

### Effect of the loss tangent

**Figure 8:**
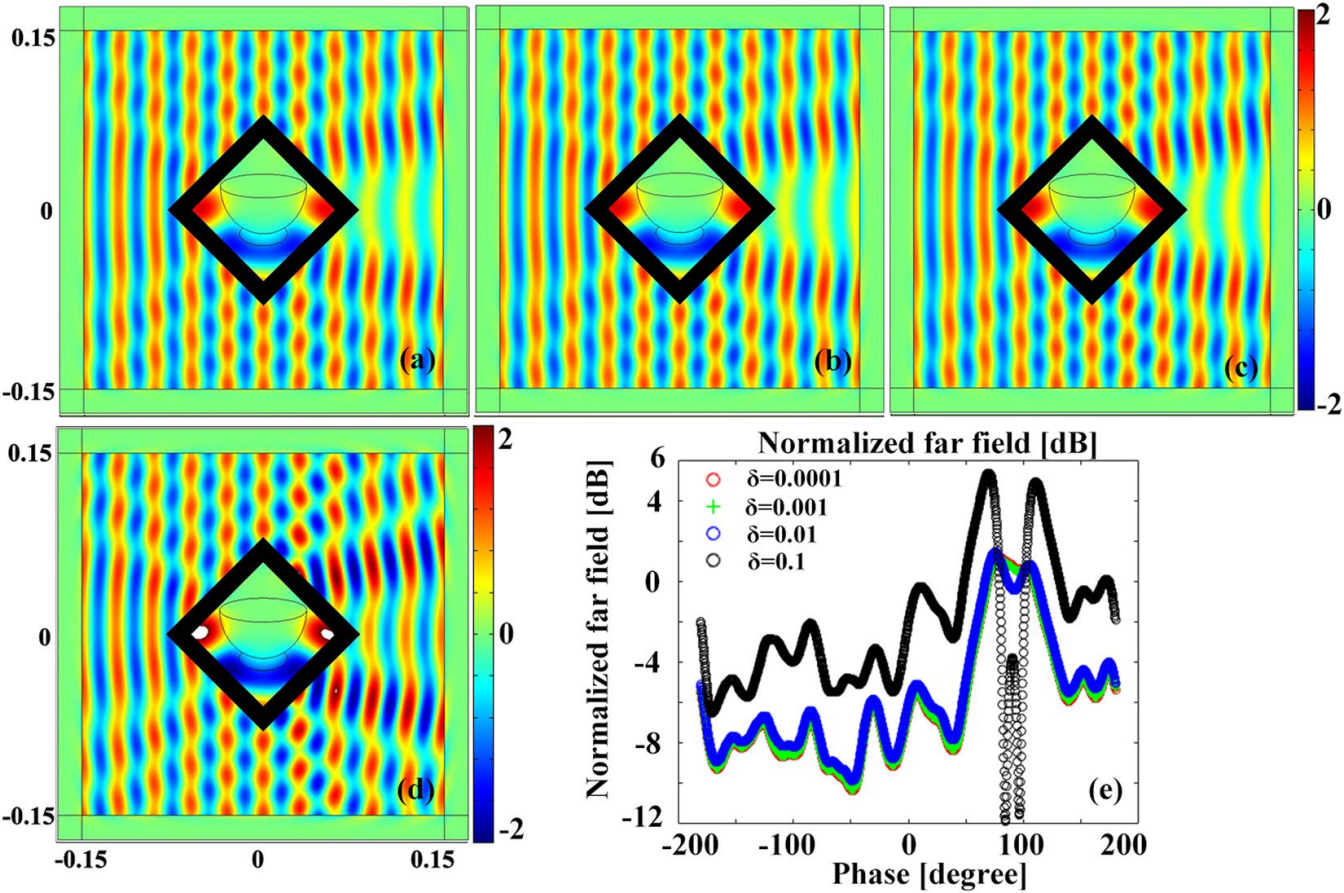
The electric field distributions of layered rotating shrink device in Fig. 6(c) when loss tangent of the layered isotropic medium is (**a**) 0.0001, (**b**) 0.001, (**c**) 0.01 and (**d**) 0.1. (**e**) The normalized far filed.

Considering metamaterials are always lossy and narrow-banded, it is necessary to investigate the effects of loss on the proposed layered structure devices. For universal and brevity, we take the layered rotating shrinking device as example and demonstrates the near/far field distributions of the layered structure device in Fig. 6(c) when the alternatively layered isotropic medium have different loss tangents. Comparing Fig. 8(a)–(d), it can be found that the near electric field distribution appealed almost identical to the ideal layered one in Fig. 6(c) when the loss tangent are 0.0001, 0.001 and 0.01. However, the electric filed distribution is distorted when the loss tangent is about 0.1. To quantitatively evaluate the performance of different loss on the proposed layered multifunctional device, the corresponding normalized far-field distributions in Fig. 8(a–d) are investigated, and the results are shown in Fig. 8(e). It is clear that the scattering pattern of the proposed layered rotating shrink device are overlapped when the loss tangent is less than 0.01. In order to obtain the proposed multifunctional devices with layered structure, the loss tangent should be smaller than 0.01 in practical engineering.

## Discussion

In conclusion, we propose a new approach to design multifunctional rotary medium with homogeneous and anisotropic material parameters based on linear transformation optics. Four kinds of multifunctional devices have been reported. Rotary concentrator can enhance the EM energy density in the core region of the device while having the field been rotated at the same time, which has potential application in energy accumulation or store. Rotary amplifying device can rotate and amplify arbitrary object into another bigger one that of different sizes and material parameters, while the rotary shrinking device has the contrary performance. Rotary transparent device can rotate the electric field while keeping the device itself transparent and invisible. All these multifunctional devices may have potential applications in military camouflage or wireless communication systems. Furthermore, alternating layered structure based on effective medium theory has been utilized to remove the anisotropic properties of these devices, which produce a strategy to implement these devices by natural homogeneous and isotropic materials instead of metamaterials. We hope our work are helpful for speeding up the designing, fabrication and applications of multifunctional devices.
